# *Triatoma brasiliensis* Neiva, 1911: food sources and diversity of *Trypanosoma cruzi* in wild and artificial environments of the semiarid region of Ceará, northeastern Brazil

**DOI:** 10.1186/s13071-018-3235-4

**Published:** 2018-12-17

**Authors:** Claudia Mendonça Bezerra, Silvia Ermelinda Barbosa, Rita de Cássia Moreira de Souza, Carla Patrícia Barezani, Ricardo Esteban Gürtler, Alberto Novaes Ramos, Liléia Diotaiuti

**Affiliations:** 1 Universidade Federal do Ceará, Departamento de Saúde Comunitária, Faculdade de Medicina, Fortaleza, CE Brasil; 2 Grupo Triatomíneos, Instituto René Rachou, Fundação Oswaldo Cruz, Belo Horizonte, Minas Gerais Brasil; 3 Secretaria da Saúde do Estado do Ceará, Fortaleza, CE Brasil; 4 Universidad de Buenos Aires, Instituto de Ecología, Genética y Evolución, Buenos Aires, Argentina

**Keywords:** Chagas disease, *Triatoma brasiliensis*, *Trypanosoma cruzi*, Discrete typing unit, Eating behavior, Caatinga, Brazil

## Abstract

**Background:**

Knowledge of triatomine food sources in different ecotopes enables the estimation of *T. cruzi* transmission risk in diverse environments, as well as its dynamics of dispersion and ecological niche. For *Triatoma brasiliensis* in the Caatinga, in the northeast of Brazil, seasonal differences influence feeding eclecticism and rates of *T. cruzi* infection. The objective of the present study was to monitor food sources and to characterize the populations of *T. cruzi* associated with *T. brasiliensis* in wild and domestic environments in the Caatinga of northeast Brazil.

**Methods:**

A cross-sectional study based on a search for triatomines in wild and domestic environments, was undertaken at five different time periods from 2009 to 2015. Insects from 2015 were used for identification of food sources. Two universal primers, based on the conserved regions of the *12S* rRNA locus, were used to amplify fragments of 215 bp. The content of the intestinal tract of triatomines was identified by a comparison between the sequences obtained and those deposited in the GenBank database, using BLAST. In triatomines with parasitological diagnosis of infection by trypanosomatids, xenoculture was performed for the isolation and characterization of strains, using *cox*2, the amplification of the SL-IL mini-exon intergenic spacer and the polymorphism of the D7 divergent domain of the gene 24αrDNA-*LSU*.

**Results:**

Food sources were identified in 76.3% (213/279) *T. brasiliensis* specimens sampled in 2015. The most frequent sources in a total of 20 vertebrate species were: rodents (58%, 123/213), ruminants (30%, 64/213) and cats (6%, 12/213). A total of 49% (44/89) of the samples of *T. cruzi* isolated in the period from 2009 to 2015 were characterized: TcII (43%, 19/44), TcI (41%, 18/44) and TcIII (16%, 7/44).

**Conclusions:**

The feeding eclecticism of *T. brasiliensis* shows its importance in maintaining the transmission dynamics of *T. cruzi*, with evidence of intense circulation between anthropic and wild environments. Attention should be placed on the association among *T. brasiliensis*, rodents and ruminants, in addition to the presence of TcIII in the study region.

**Electronic supplementary material:**

The online version of this article (10.1186/s13071-018-3235-4) contains supplementary material, which is available to authorized users.

## Background

Chagas disease is a neglected chronic infectious disease that persists with high rates of morbidity and mortality. Approximately 6–7 million people are infected worldwide and the disease causes 12,000 deaths/year [1–3]. Its magnitude and transcendence reinforces the fact that it is a priority as a public health problem in Brazil [3].

*Trypanosoma cruzi* (Protozoa: Sarcomastigophora: Kinetoplastida: Trypanosomatidae) [4], the etiological agent of Chagas disease, has high adaptive success, with different degrees of tissue tropism, virulence and susceptibility to drugs. It has a broad host range which includes more than 150 species of mammals. It can colonize virtually any tissue of these vertebrates, and it may be transmitted by dozens of species of triatomines (Hemiptera: Reduviidae: Triatominae) [5–8].

*Trypanosoma cruzi* is composed of heterogeneous populations classified into seven DTUs (discrete typing units): TcI-TcVI [9, 10] and TcBat, which are associated with bats and genetically similar to TcI [11]. The first six DTUs can also cause infections and diseases in humans. However, the prevalence and dispersion of these DTUs differ according to geographical and ecological niches, with variations in clinical epidemiology [9, 12].

Triatomines, obligatory hematophagous hemipterans of the family Reduviidae (subfamily Triatominae), are subdivided into five tribes and more than 150 species [13]; of these, 65 (44%) occur in Brazil [14, 15]. In the Caatinga, an exclusively Brazilian biome, there is a high triatomine diversity, with 18 (27%) of the species recorded in Brazil [15–17]. Theoretically, all triatomine species are considered capable of transmitting the six *T. cruzi* lineages described [18], participating in the maintenance of the enzootic cycle or domestic cycles [19, 20].

Three subspecies of *Triatoma brasiliensis* have been recognized [21]: *T. brasiliensis brasiliensis* Neiva, 1911; *T. brasiliensis melanica* Neiva & Lent, 1941; and *T. brasiliensis macromelasoma* Galvão, 1956. In 1979, Lent & Wygodzinsky [22] considered the three subspecies as synonyms for *T. brasiliensis* and claimed that the differences among them were only chromatic. In 2006, the subspecies *T. b. melanica* was elevated to the species rank as *Triatoma melanica* [23]. The same occurred with *Triatoma juazeirensis* in 2007 [24], cited by Lent & Wygodzinsky [22] as a dark variant of *T. brasiliensis*. Thus, the *T. brasiliensis* complex currently includes two subspecies (*T. brasiliensis brasiliensis* and *T. b. macromelasoma* [25]) and six species (*T. lenti*, *T. juazeirensis*, *T. melanica*, *T. bahiensis* [26], *T. sherlocki* [27] and *T. petrocchiae* [14, 28].

*Triatoma brasiliensis brasiliensis* Neiva, 1911, hereby referred to as *Triatoma brasiliensis*, is the main species responsible for *T. cruzi* transmission in the Northeast region of Brazil. Its center of dispersion is related to the Caatinga biome [29]. In wild environments (particularly in rock outcrops) there are colonies with high infection rates associated with several species of bats, marsupials and rodents [30–32]. In sedimentary plains, these insects can be associated with the cactus *Pilosocereus gounellei* [33].

From the recognized geographical distribution of *T. brasiliensis*, analyses of usual entomological indicators showed the epidemiological importance of the species in Bahia (BA), Ceará (CE), Piauí (PI), Paraíba (PB), Pernambuco (PE) and Rio Grande do Norte (RN) states, emphasizing their high rates of intradomicile infestation, high population density and variable percentages of natural infection by trypanosomatids [34, 35].

In Ceara State, *T. brasiliensis* was initially recognized in 1922 by Neiva & Pinto, in the Jaguaribe mesoregion. From 1955 to 1983, this species was found present in 86% (121/141) of the municipalities of Ceará, with 44% (62/141) of specimens infected with *T. cruzi* [30]. Technical reports from the Health Department of Ceará State show that between 2000–2017, 289,907 specimens of *T. brasiliensis* were captured in 87% (161/184) of the municipalities in the state, with natural infection with trypanosomatids in 65% (119/184) of these areas.

In extreme natural conditions, in order to obtain a blood meal, *T. brasiliensis* exceeds microclimatic preferences established in the laboratory [36], and it is able to fiercely attack humans and animals, even during daylight [37, 38]. Feeding eclecticism and opportunism are striking characteristics of triatomines, which can suck blood from a wide variety of vertebrates [39]. Understanding these aspects in anthropic environments can support strategies for vector control, especially in species such as *T. brasiliensis*, which represent an important operational challenge. This fact occurs in view of the constant recolonization of intra- and peridomiciles that can occur as a result of natural foci or specimens which remain after chemical control [36, 37, 40–43].

Food availability is a determinant factor for the size of triatomine populations in various ecotopes [44]. In this context, the anthropic environment provides not only a great availability of food sources but also a wide range of shelter. For this reason, survivors of the peridomiciliar colonies are frequent, contributing to reinfestation of the domestic environments [32].

The feeding eclecticism of *T. brasiliensis* has already been reported in different studies [45, 46]. This species is capable of feeding on humans, dogs and cats in artificial environments or natural ecotopes [30]. Blood of rodents, reptiles, mammals and birds were identified as food sources in samples of the stomach contents of wild *T. brasiliensis* specimens [47]. There have also been reports that seasonal differences influence feeding eclecticism as well as rates of infection with *T. cruzi* [48]. The latter study found a decrease in the quantity of food sources identified when droughts lasted longer. Another finding was that the main food sources of *T. brasiliensis* were birds (33.1%) and armadillos (18.8%). The extensive farming of chickens and goats in the Brazilian state of Ceará was highlighted as a factor of connection between anthropic and wild environments [49]. DNA sequences of chickens (50%) and goats (29%) were identified in samples of the stomach contents of triatomines from a wild environment, since they are often found in these environments. Thus, knowledge of triatomine food sources in different ecotopes assists in estimating the risk of transmission of *T. cruzi* in diverse environments, as well as its dynamics of dispersion and ecological niche [40, 50]. In this perspective, the objective of the present study was to monitor food sources and to characterize *T. cruzi* populations associated with *T. brasiliensis* in wild, intradomicile and peridomicile environments in the Caatinga, in Tauá municipality of Ceará State, in the Northeast Brazil region.

## Methods

### Research design and study site

**Fig. 1:**
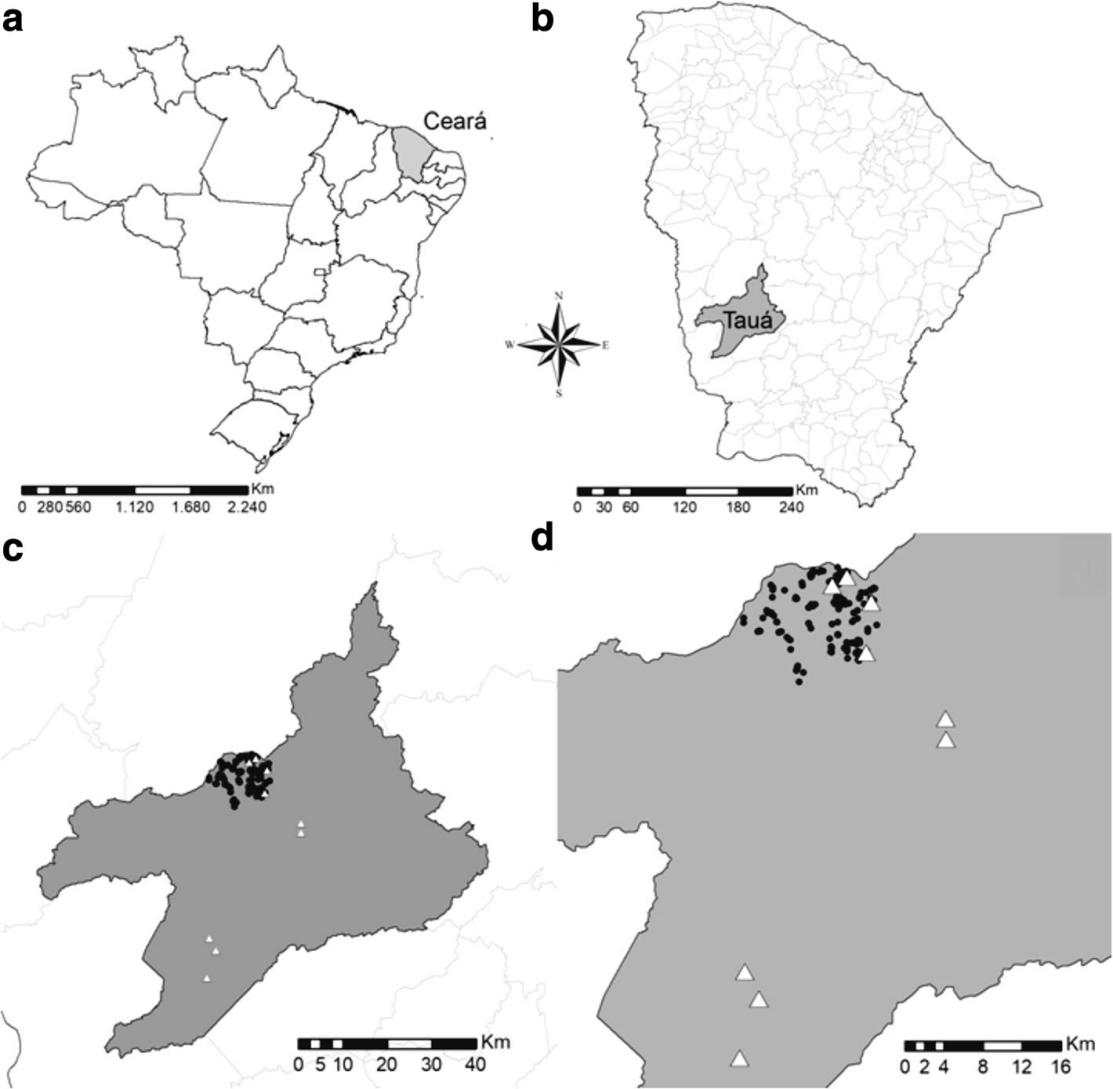
**a** Location of Ceará State, Brazil. **b** Area of Tauá munipality, Ceará, Brazil. **c** Study site. **d** Detail of study site. Circles indicate anthropic environments; triangles indicate wild environments. Source: adapted from Google Earth and QGis 2.14. Essen

This is a descriptive ecological study conducted in Tauá municipality, in Ceará State (Fig. 1), a region of epidemiological and historical importance in the context of the vectorial transmission of Chagas disease, with high levels of infestation by triatomines, and predominance of *T. brasiliensis* [34, 49, 51].

Tauá is located in the hinterland of Inhamuns (6°00'11"S, 40°17'34"W) at an altitude of 402.7 m, 320 km from Fortaleza. The average annual temperature ranges from 26 °C to 28 °C and the average annual rainfall is 597.2 mm^3^, with the rainy period between February and April [52].

The shrub-arboreal Caatinga is the predominant vegetation, especially thorny deciduous vegetation, associated with rock soils with expressive fractures and extreme water deficiency in for most of the year [53]. The granite rocks provide shelter to small mammals, reptiles and insects, including *T. brasiliensis*, which has this environment as its natural habitat [29].

A search for triatomines was performed in 252 housing units (HU) of 18 rural localities (Fig. 1), where the last residual chemical control for triatomines had occurred 24 months before. These HUs were investigated in five periods (February 2009, August 2009, March 2010, October 2010 and August 2015), in order to ensure representativeness of the seasonality which is typical of Ceará State. The search was carried out manually, as advocated by the Technical Standards Manual (1980) [54], by municipal staff for the control of endemic diseases. They were supervised by the Ceará State Health Secretariat (SESA-CE) and used appropriate forms as a data collection instrument (Additional file 1). The search was performed exhaustively, i.e. through all the rooms of the households and peridomicile ecotopes, seeking to capture all insects found. HU represents the epidemiological unit of reference in vector control, formed by the whole set of the human dwelling and its surroundings with all permanent and temporary buildings, accumulations of materials, fences, animal shelters, etc. [54]. The intradomicile was considered as a single space, and the peridomicile was divided into types of annexes: (i) chicken coop; (ii) pigsty; (iii) barn; (iv) firewood; (v) stone, brick and roofing tile; and (vi) other.

The ecotopes of the triatomines captured in the peridomicile were marked, allowing the description of families of vertebrate animals, identified as food sources according to developmental instar and specific place of capture. When the peridomicile ecotopes were categorized, the importance of “roofing tiles, bricks and stones” and “firewood” was recognized. Therefore, a decision was made to include in the analysis the location of these structures in the peridomicile. Records were made whether they were within the structures built to house economically important synanthropic animals, for example, “pens”, or if they were randomly dispersed in the environment.

In parallel and within the corresponding area to the search of infestation in the HU, the search for wild triatomines populations was conducted in three areas with rock formations that could be natural shelters of *T. brasiliensis*. These areas are located at variable distances from human dwellings. In the search conducted in August 2015, the wild capture area was expanded to other six places in the municipality with similar characteristics to those of the previously defined areas (Fig. 1).

### Laboratory tests

Captured triatomines were initially identified to the species level [22, 25], place of capture and developmental instar. Fresh feces, obtained by abdominal compression, were examined to determine infection with trypanosomatids with an optical microscope (400×). The intestinal contents of most positive triatomines were placed in culture medium for the isolation of strains [55].

Food sources were identified by using insects captured in August 2015. They were kept in cryogenic vials containing 70% ethyl alcohol and frozen at -20 °C until analysis. Procedures for parasitological diagnostic of feces were conducted in the Laboratories of Entomology Dr Tomaz Aragão, Ceará State, Health Secretariat (SESA-CE) and the Reference Laboratory in Triatomines and Epidemiology of Chagas Disease of the René Rachou Institute (IRR)/Fiocruz Minas. Molecular characterization of parasites and determination of food sources of the triatomines were performed by the IRR.

### Identification of food sources

The extraction of stomach contents used only adult insects and fifth-instar nymphs. The remaining stages were not used because the insects had little blood in the digestive tube and thus a low chance of presenting conclusive results. For exposure of the digestive tube, insects were dissected; the wings of adults were removed, and in both stages, the conexivum was removed, thus allowing the separation of the dorsal and ventral cuticles of the abdomen of the insects. Digestive tube was separated and placed in microtubes containing 70% ethanol. It should be noted that the stomach contents of the insects were only removed if, when dissected, they clearly showed food contents in the digestive tract.

### PCR amplification and direct sequencing of the *12S* rRNA locus

Total DNA extraction from triatomine digestive tract content used the protocol of the DNeasy Blood and Tissue Kit™ (Qiagen, Hilden, Germany). Two universal primers for vertebrate animals designed based on the conserved regions of the *12S* locus of the rRNA (L1085 5'-CCC AAA CTG GGA TTA GAT ACC C-3' and H1259 5'-GTT TGC TGA AGA TGG CGG TA-3') which amplified a fragment of 215 bp [56].

PCR was performed in a final volume of 25 μl, containing 40–50 ng of genomic DNA, 2.5 μl of buffer 10×, 2.5 μl of dNTP 2.5 Mm, 0.75 μl of MgCl_2_ 50 mM, 2.5μl of each primer (10 pmol) and 0.25 μl of Taq Platinum 0.5U/μl (Invitrogen, California, USA). For each PCR reaction, a negative control (without DNA) was run in parallel.

PCR was conducted in 35 cycles of 95 °C for 30 s, 57 °C for 15 s and 72 °C for 30 s using a Veriti™ thermal cycler (Applied Biosystems, Foster City, CA, USA). Amplified products were observed in 8% polyacrylamide gel stained with silver nitrate 0.2%.

The products of the positive samples after PCR were purified using a QIAquick PCR Purification Kit™ (Qiagen), according to the manufacturer’s protocol. Purified PCR products were sequenced using a BigDye Terminator v.3.1 Cycle Sequencing Kit™ and an ABI 3730XL DNA Analyzer™, both from Applied Biosystems.

### Identification of sequences

To identify the food sources of the triatomines being analyzed, the resulting sequences were compared with sequences deposited into the GenBank database by using the search tool through the software BLASTn (https://blast.ncbi.nlm.nih.gov/Blast.cgi).

### Characterization of *T. cruzi* according to DTUs

#### Samples

After the parasitological diagnosis had been confirmed, parasites were kept in liquid culture (liver infusion tryptose, LIT). The samples that showed fungal contamination were discarded. The others were maintained in a state of exponential growth to obtain approximately 10^5^ parasites/ml of culture. DNA was extracted by the phenol-chloroform method as described by Vallejo et al. [57].

#### Triple assay

The classification of DNA samples of *T. cruzi* was based on the protocol proposed by Davilla et al. [58]. This scheme proposes a classification considering the mitochondrial gene cytochrome *c* oxidase subunit 2 (*cox*2) [59], the amplification of the mini-exon intergenic spacer of *T. cruzi* [60], and the polymorphism of the D7 divergent domain of the gene 24αrDNA - *LSU* rDNA [61]. All reactions were carried while using positive and negative controls. Fragments generated by the amplification of the genes 24αrDNA and *cox*2 were visualized on polyacrylamide gel at 6% with silver staining. The amplified product from the mini-exon was visualized on 2% agarose gel. Gel Red Nucleic Acid Stain 1:300 (Biotium, Fremont, CA, USA) was used as a fluorescent marker of the amplified band.

### Spatialization of *T. cruzi* populations

Geographical coordinates of the positive ecotopes for the presence of triatomines were recorded along the activities with the aid of a Garmin eTrex™ 12-channel GPS, with WGS89 - Zone 24S projection. Subsequently, points were geocoded in high resolution, in accordance with the basis of Google Earth Pro® software v.7.1, generating a map embedded in the environment of the geographical information system (GIS). After that, the exploratory analysis of spatial behavior of events was based on the estimated kernel density to create a *raster* map whereby the density was based on the number of points in the study region. In this way, it was checked whether the events occurred at random or there were aggregations among them (*hotspots*), indicating the occurrence of clusters [62]. For the thematic map of the distribution of *T. cruzi* populations, a radius of 700 m was considered, generated in the software QGis v.2.14, an open-source geographical information system (https://qgis.org/en/site/about/index.html).

### Statistical analysis

Rates of infestation and colonization were calculated based on the housing units (intradomicile and peridomicile) with the presence of triatomines in the searched units. Natural infection refers to triatomines parasitized by trypanosomatids in the examination of fresh feces. The association among habitat, blood source (or host) and parasite genotype was checked by using Fisher’s exact test and Pearson’s test, with a statistical significance of 0.05%. The analyses were performed in the software Stata v.15.1. (StataCorp LP, College Station, TX, USA).

## Results

The results of the five catches in the study period are shown in Additional file 2: Table S1; 66.4% (1928/2906) of the triatomines found in the housing units were identified as *T. brasiliensis* and 33.4% (971/2906) as *Triatoma pseudomaculata*. In the wild environment, 1077 *T. brasiliensis* specimens were captured. The natural infection indices found for trypanosomatids were: intradomicile (10.3%, 18/175); wild environment (3.3%, 32/979); and peridomicile (3.1%, 51/1629).

### Food sources

Of the 279 samples processed for the identification of the food source of triatomines, 194 (69.5%) presented identity greater than or equal to 95% when compared to the sequences in GenBank. Identities of 90–94% were found in 19 (8.6%) samples, in a total of 213 samples (76.3%) with satisfactory results. Most of these triatomines (46.5%, 99/213) were captured in the peridomicile. The developmental instar with the highest representation in the sample was nymph N5 (35.2%, 75/213) (Table 1; Additional file 2: Tables S2 and S3). Twenty species of animal were distributed into 13 families, of which 10 (76.9%) were from wild triatomines, 10 (76.9%) from peridomicile and 9 (69.2%) from intradomicile environments (Table 2; Additional file 2: Table S3).

**Table 1:** Number of *Triatoma brasiliensis* sampled for characterization of food sources in accordance with place of capture and developmental stage, in Tauá municipality, Ceará, Brazil, 2015

Environment	Nymph 5 *n* (%)	Male *n* (%)	Female *n* (%)	Total *n* (%)
Peridomicile	27 (27.3)	28 (28.3)	44 (44.4)	99 (100)
Wild	46 (53.5)	24 (27.9)	16 (18.6)	86 (100)
Intradomicile	2 (7.1)	17 (60.7)	9 (32.1)	28 (100)
Total	75 (35.2)	69 (32.4)	69 (32.4)	213 (100)

**Table 2:** Food sources of *Triatoma brasiliensis* in the anthropic and wild environments, identified by PCR with primers (L1085 and H1259) projected with a basis on the conserved regions of the *12S* rRNA locus, in Tauá municipality, Ceará, Brazil, 2015

Class	Order	Family	Species	GenBank ID	Associated environment
Mammalia	Rodentia	Echimyidae	*Proechimys cuvieri*	KU892778.1	W, I, P
			*Proechimys roberti*	KU892772.1	
			*Thrichomys apereoides*	KU892773.1	
		Caviidae	*Galea spixii*	AF433913.1	W, I, P
			*Kerodon rupestris*	AY765988.1	
		Cricetidae	*Wiedomys cerradensis*	KF769457.1	W, I, P
			*Oecomys bicolor*	KX381448.1	
		Muridae	*Rattus rattus*	KX381445.1	P
			*Mus musculus*	KX381752.1	
	Artiodactyla	Bovidae	*Bos taurus*	KT343749.1	W, I, P
			*Capra hircus*	KY305183.1	
		Suidae	*Sus scrofa*	KT194220.1	I, P
		Capridae	*Ovis aries*	KR868678.1	W, I, P
	Didelphimorphia	Didelphidae	*Monodelphis domestica*	AJ508398.1	W, I
	Perissodactyla	Equidae	*Equus cabalus*	KX669268.1	I
	Carnivora	Felidae	*Felis silvestris*	KX002032.1	W, I, P
Aves	Psittaciformes	Psittacidae	*Pionus menstruus*	KX925978.1	W, P
			*Forpus crassirostris*	DQ143215.1	
	Galliformes	Phasianidae	*Meleagris gallopavo*	JF275060.1	W, P
			*Gallus gallus*	KX781319.1	
Reptilia	Squamata	Phyllodactylidae	*Phyllopezus pollicaris*	KJ484234.1	W

*Abbreviations*: *W* wild, *I* intradomicile, *P* peridomicile

Although rodents (57.8%, 123/213) and goats (21.1%, 45/213) were the most frequent food sources in all environments, there was no statistically significant difference between the animal group identified as a food source and the ecotope where triatomine was captured (Pearson’s Chi-square test: *χ*^2^ = 11.3801, *df* = 6, *P* = 0.77; Fisher’s exact test: *P* = 0.06) (Table 3). In addition to these two groups, cattle and cats were also identified in the study environments. Of the seven groups of animals identified in the wild and intradomicile environments, five were coincident: rodents, goats, cats, cattle and marsupials. DNA samples of horses and pigs were identified in the intradomicile; they were both related to adult *T. brasiliensis* males. The intradomicile sample of marsupial DNA belonged to a female *T. brasiliensis* specimen. Of the groups of animals characterized in the peridomicile and wild environment, blood samples of marsupials and reptiles were present in triatomines, while pigs were characterized only for insects found in the peridomicile. In the housing units, birds were characterized only in the peridomicile, while marsupials and horses were only described in the intradomicile. The only human DNA sample was recorded in a nymph N5 in the peridomicile, but its similarity was 88% when compared with sequences on GenBank; therefore, it was regarded as negative (Table 3).

**Table 3:** Animals identified as food sources of *Triatoma brasiliensis* according to capture environment, Tauá, Ceará, Brazil, 2015

Animal group	Intradomicile *n* (%)	Peridomicile *n* (%)	Wild *n* (%)	Total *n* (%)
Rodents	15 (54)	53 (54)	55 (64)	123 (58)
Goats	5 (18)	27 (27)	13 (15)	45 (21)
Cattle	3 (11)	12 (12)	4 (5)	19 (9)
Cats	2 (7)	2 (2)	8 (9)	12 (6)
Birds	–	4 (4)	2 (2)	6 (3)
Marsupials	1 (4)	–	3 (3)	4 (2)
Pigs	1 (4)	1 (1)	–	2 (1)
Horses	1 (4)	–	–	1 (0)
Reptiles	–	–	1 (1)	1 (0)
Total	28 (100)	99 (100)	86 (100)	213 (100)

Pearson’s Chi-square test: *χ*^2^ = 11.3801, *df* = 6, *P* = 0.77; Fisher’s exact test: *P* = 0.06

### Ecotopes

Triatomines captured in “roofing tiles, bricks and stones” accounted for the largest part of the sample (54.5%), as well as for the greatest diversity of families of vertebrate animals identified as a food source. Adult triatomines were those which had the largest number of food sources identified (*n* = 72), present in all peridomicile ecotopes described in this study. Nymphs N5 were sampled at only four ecotopes, but they had greater diversity of associated food sources (*n* = 6) (Table 4). For the purpose of analysis, animal groups were simplified to rodents, goats and other (sum of other groups). The same procedure was adopted for the sampled triatomines, i.e. nymphs and adults were grouped together. The analysis showed that there is a statistically significant difference between the group of animals identified as a food source in comparison to the peridomicile ecotope in which triatomines were caught (Pearson’s Chi-square test: *χ*^2^ = 17.8224, *df* = 8, *P* = 0.023; Fisher’s exact test: *P* = 0.02). Rodents are the most expressive food sources in roofing tiles, bricks and stones while rodents and goats are equally important in “sheepfolds”.

**Table 4:** Food sources of *Triatoma brasiliensis* by developmental stage and ecotopes of capture in peridomicile environments, Tauá, Ceará, Brazil, 2015

Ecotope	Rodents, *n* (%)		Goats, *n* (%)		Birds, *n* (%)		Cats, *n* (%)		Cattle, *n* (%)		Pigs, *n* (%)	Subtotal, *n* (%)		Total *n* (%)
	Adults	N5	Adults	N5	Adults	N5	Adults	N5	Adults	N5	N5	Adults	N5	
Roofing tiles, bricks and stones	27 (81.8)	12 (57.1)	8 (9.1)	5 (23.8)	0 (0)	1 (4.8)	1 (3.0)	1 (4.8)	2 (6.1)	1 (4.8)	1 (4.8)	38 (61.1)	21 (38.9)	59 (54.5)
Sheepfold	4 (36.4)	1 (100)	5 (45.5)	0 (0)	1 (9.1)	0 (0)	0 (0)	0 (0)	1 (9.1)	0 (0)	0 (0)	11 (91.7)	1 (8.3)	12 (12.1)
Firewood	6 (66.7)	0 (0)	2 (33.3)	3 (100)	1 (0)	0 (0)	0 (0)	0 (0)	4 (0)	2 (0)	0 (0)	13 (66.7)	5 (33.3)	18 (9.1)
Chicken coop/perch	2 (25.0)	–	1 (25.0)	–	1 (0)	–	0 (0)	–	2 (50.0)	–	–	6 (100)	0 (0)	6 (4.0)
Pigsty	1 (25.0)	–	3 (75.0)	–	0 (0)	–	0 (0)	–	0 (0)	–	–	4 (100)	0 (0)	4 (4.0)
Total	40 (55.6)	13 (48.1)	19 (26.9)	8 (29.6)	3 (4.7)	1 (3.7)	1 (1.9)	1 (3.7)	9 (12.5)	3 (11.1)	1 (3.7)	72 (72.7)	27 (27.3)	99 (100)

Pearson’s Chi-square test: *χ*^2^ = 17.8224, *df* = 8, *P* = 0.023; Fisher’s exact test: *P* = 0.02

### Characterization of *T. cruzi*

Altogether, 101 triatomines had been infected with trypanosomatids, as shown by parasitological examination of fresh feces, of which 87.1% (88/101) had isolated parasites, of which 48.9% (43/88) were characterized. A sample of TcIII was characterized after the capture of *M. domestica* [47] (1/88) (Table 5).

**Table 5:** Characterization of *Trypanosoma cruzi* using the amplification of *cox*2, of the mini-exon intergenic spacer (SL-IL) and polymorphism of the D7 divergent domain of the gene 24αrDNA-*LSU* rDNA, Tauá municipality, Ceará, Brazil, 2009 to 2015

Period	Place of capture	Ecotope	Associated species	No. of samples	TcI	TcII	TcIII
February 2009	Peridomicile	Bricks	*T. brasiliensis*	6	–	6	–
		Stones	*T. brasiliensis*	1	–	1	–
		Firewood	*T. brasiliensis*	5	5	–	–
	Wild	Stones	*T. brasiliensis*	2	–	1	1
	*n*			14	5	8	1
August 2009	Wild	Stones	*T. brasiliensis*	6	–	6	–
	*n*			6	–	6	0
March 2010	Intradomicile	Intradomicile	*T. brasiliensis*	2	1	–	1
	Peridomicile	Roofing tiles	*T. brasiliensis*	2	1	1	–
		Sheepfold	*T. brasiliensis*	1	1	–	–
		Chicken coop	*T. brasiliensis*	2	–	2	–
	Wild	Stones	*Monodelphis domestica* ^a^	1	–	–	1
	*n*			8	3	3	2
October 2010	Intradomicile	Intradomicile	*T. brasiliensis*	1	1	–	–
	Peridomicile	Pigsty	*T. brasiliensis*	4	4	–	–
		Roofing tiles	*T. brasiliensis*	3	2	1	–
	*n*			8	7	1	–
August 2015	Intradomicile	Intradomicile	*T. brasiliensis*	1	–	–	1
	Peridomicile	Chicken coop	*T. pseudomaculata*	1	–	1	–
		Roofing tiles	*T. brasiliensis*	2	2	–	–
		Firewood	*T. brasiliensis*	1	1	–	–
	Wild	Stones	*T. brasiliensis*	3	-	-	3
	*n*			8	3	1	4
	*N*			44	18	19	7

^a^Bezerra et al. [47]

*Abbreviations*: TcI *T. cruzi I, TcII T. cruzi* II, *TcIII T. cruzi* III, *n*, subtotal: *N*, total

Of the discarded samples, 51.1% (45/88) had been contaminated by fungi, while in 12.8% (13/101) of triatomines with positive parasitological examination, the parasite could not be isolated because it did not have enough intestinal content for the purposes of culture. The poor nutritional status of triatomines hampered the identification of the food source of those which were infected.

**Fig. 2:**
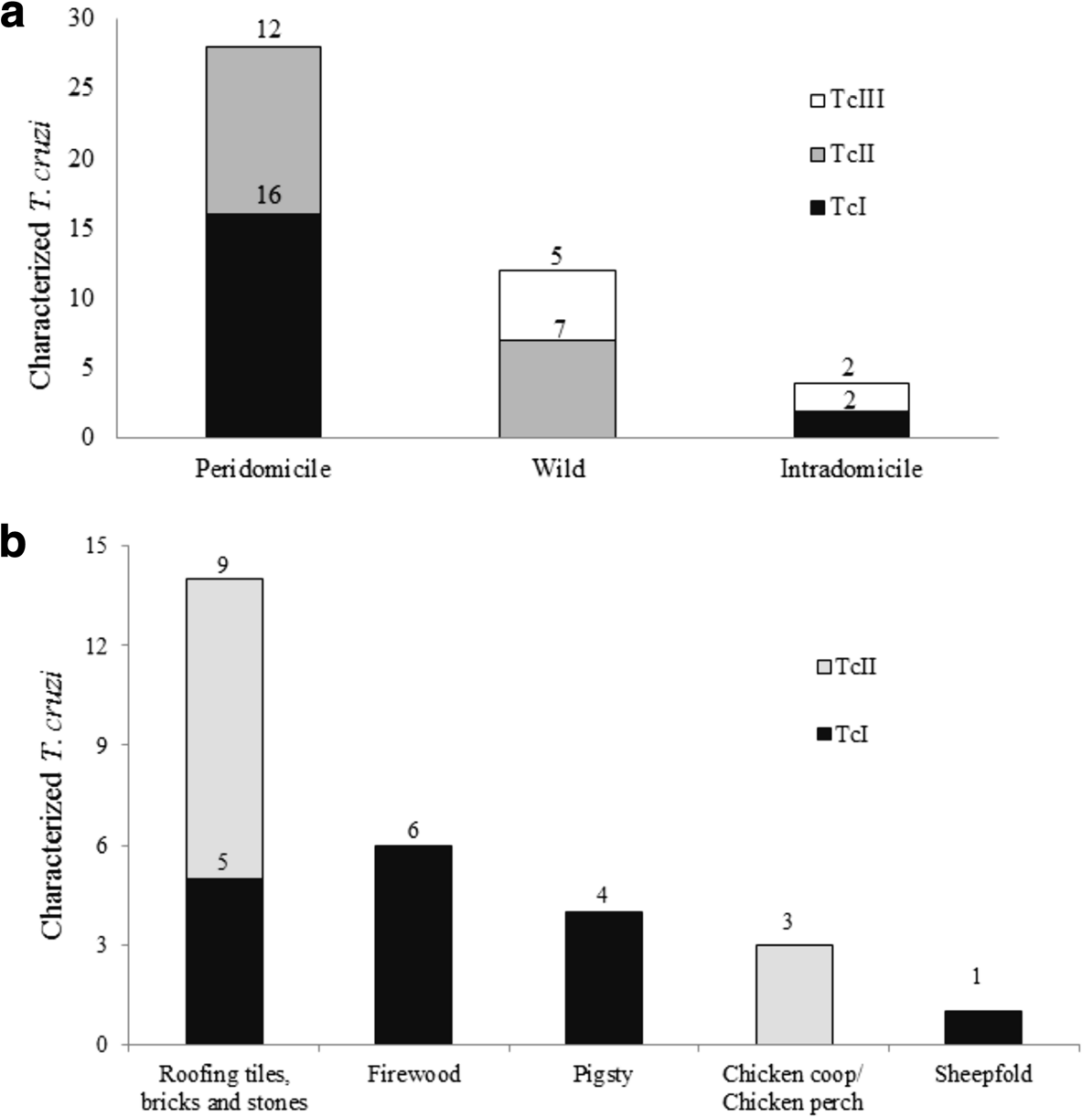
Prevalence of *Trypanosoma cruzi* (TcI: *T. cruzi* I; TcII: *T. cruzi* II; TcIII: *T. cruzi* III) in *Triatoma brasiliensis*, *T. pseudomaculata* and *Monodelphis domestica* caught in different environments in an area of the Caatinga, Tauá municipality Ceará, Brazil, 2009 to 2015. **a** Total number of *T. cruzi* characterized by DTU and environment of origin. **b** Characterization of *T. cruzi* by DTU according to peridomicile ecotopes of origin

**Fig. 3:**
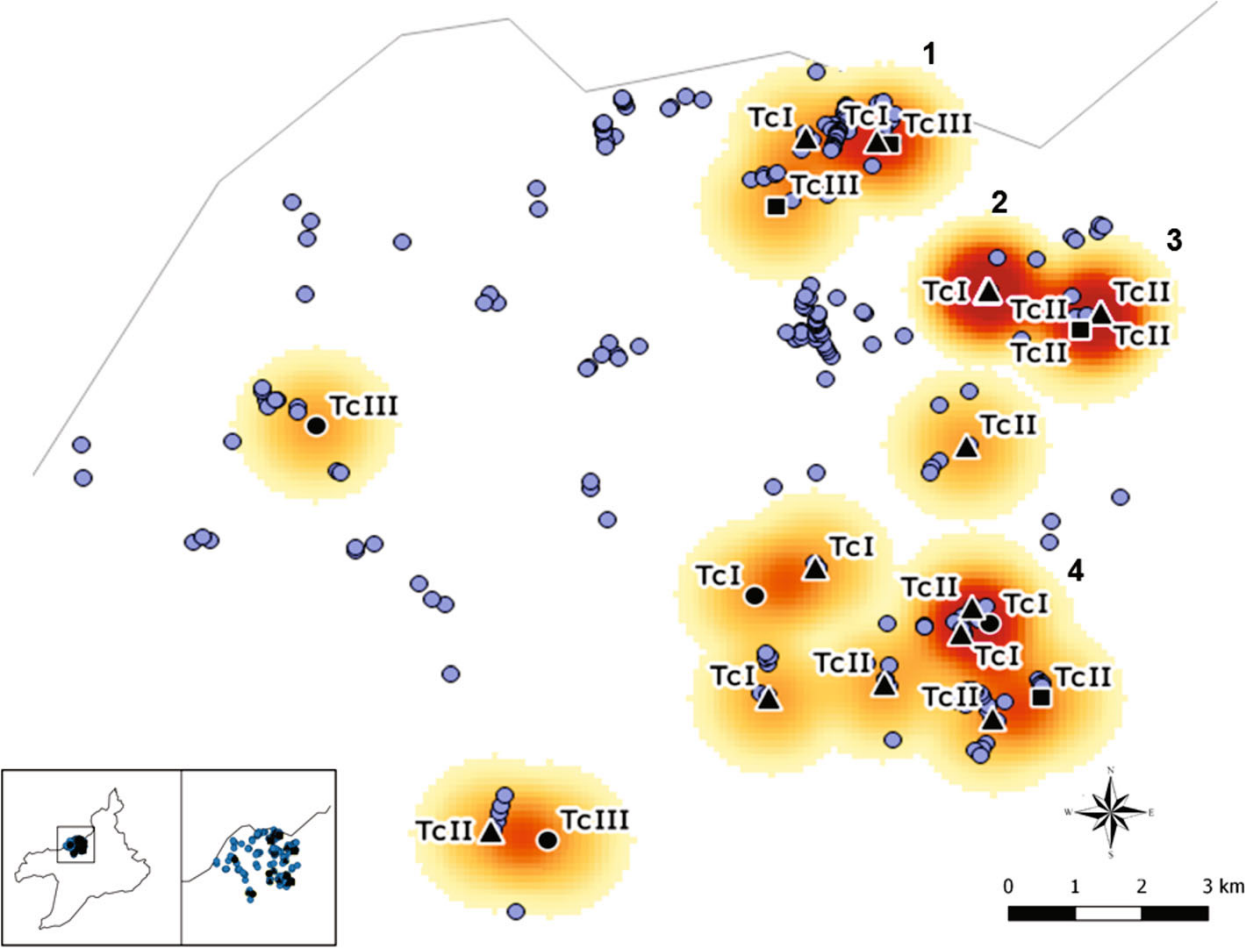
Map showing the spatial distribution (kernel) of populations of *Trypanosoma cruzi* characterized in Tauá municipality, Ceará, Brazil, from 2009 to 2015. Blue circles indicate study housing units; black circles indicate intradomicile environments; triangles indicate peridomicile environments; squares indicate wild environments. *Abbreviations*: TcI, *T. cruzi* I; TcII, *T. cruzi* II; TcIII, *T. cruzi* III

The environment of housing units had the largest number of characterized *T. cruzi* populations (73%, 32/44); peridomicile accounted for 63.6% (28/44) of the samples. “Roofing tiles, bricks and stones” corresponded to peridomicile ecotopes which had the highest number of characterized *T. cruzi* populations (50%, 14/28). In total, 43.2% (19/44) of the samples corresponded to TcII, 40.9% (18/44) to TcI and 15.9% (7/44) to TcIII (Table 5, Figs. 2 and 3).

Spatial analysis identified four main clusters of *T. cruzi* populations distributed in different environments (Fig. 3). Peridomicile contributed to the formation of all of them. TcI was described in clusters 1 (roofing tiles), 2 (pigsty, roofing tiles and firewood) and 4 (sheepfold). In addition, in the peridomicile, TcII was found in clusters 3 (bricks) and 4 (chicken coop). Wild environment (stones) contributed in clusters 1 (TcIII) and 3 (TcII). Intradomicile has participated in the formation of cluster 4, with the TcI population of *T. cruzi*. This way, the following parasites were identified: those characterized as TcI and TcIII in the intradomicile environment, as TcI and TcII in the peridomicile, and as TcII and TcIII in the wild environment.

## Discussion

The results show a wide distribution of *T. brasiliensis* specimens in the analyzed locations and occupying natural or artificial ecotopes. The evaluation of the feeding behavior of this vector in artificial (anthropic environment) and wild ecotopes acknowledges its close relationship with rodents, goats, cats and cattle. In the samples from the wild environment, DNA was identified from ten families of vertebrates, including marsupials, birds, reptiles, goats, sheep, cattle, cats and chickens. The recognition of nymphs fed with the blood of domestic animals, in view of their limited mobility, shows the importance of these animals, which move along the wild environment as a source of food for *T. brasiliensis* in its natural ecotope.

The detection of DNA of marsupials in intradomicile triatomines signals the risk of transmission of the parasite to humans because they have recognized synanthropic activity and often present high rates of natural infection by *T. cruzi*. Historically, the species of the family Didelphidae are recognized as a “bridge” between the wild and domestic cycles of *T. cruzi* [63–67].

In most adult triatomines caught in the intradomicile, the DNA of nine families of vertebrate animals was identified, especially wild animals and economically important animals reared in semi-extensive farming systems in the peridomicile environment. The contact with different food sources demonstrates the intense mobility of *T. brasiliensis* [39], which results in the colonization of the artificial environment [68]. As for nymphs, DNA samples from cats and goats, animals which circulate in the intradomicile in the study area, were characterized. We also found triatomines that had fed on the blood of goats in the intradomicile likely because the local population rears offspring of these animals inside the household when they are abandoned by their mothers.

Three families (Echimyidae, Caviidae and Cricetidae) of wild rodents which are considered to be natural hosts of *T. cruzi* were characterized in triatomines captured in all the study environments (Table 2). Considering the food sources identified in this study and the abundance of the rodents, it can be inferred that these mammals are the main food sources of triatomines. Moreover, they probably also have the role of primary reservoir of *T. cruzi* in the region, as found in previous studies [49, 69, 70].

In addition to rodents, cats and goats/sheep represented important food sources for *T. brasiliensis* in natural and domestic ecotopes, which can also be a link between domestic and wild cycles of *T. cruzi*. In the literature, cats are recognized as an important food source of triatomines [30, 45, 69, 71]; furthermore, they maintain populations of *T. cruzi* [69]. Although goats/sheep are often cited as food sources for *T. brasiliensis* in the semi-arid Northeast region [47, 49, 65, 70, 72], there are still important knowledge gaps on the actual role of these animals in the epidemiology of Chagas disease in the region, especially if the economic representation of these animals is taken into consideration. Meat, milk, viscera and leather of these animals are marketed, often *in natura*.

The identification of *Felis silvestris* (a species that does not occur in Brazil) as a food source for *T. brasiliensis* in this study is likely due to the limitation of GenBank for the identification of sequences, and it is believed that the animal corresponds to *Felis domesticus*, which is very common in the region. Absence of specific sequences, DNA fragmentation by degradation and a description of mixed food sources are methodological gaps that still need to be overcome.

Despite the high dispersion of triatomines in evaluated environments, human DNA was not detected in the samples of the captured vectors. This result may indicate some kind of self-protection of this community to minimize the contact with triatomines; of note, numerous studies have shown the intimate and persistent relation between *T. brasiliensis* and humans [30, 32, 39, 40, 42, 43, 46, 47, 49, 70].

The peridomicile represents a complex set of ecotopes (artificial and natural) that sometimes overlap, thus forming a network of ecological niches that interact, depending on human intervention and climatic conditions. Shelters in peridomicile annexes favor the establishment and proliferation of triatomine colonies, but this phenomenon does not occur consistently in time and space because these structures are usually transient and renewable. Thus, some artificial ecotopes have been shown to be more attractive than others for the development of these insects [42, 73, 74].

The results of this study show the importance of “roofing tiles, bricks and stones”, because in addition to the abundance of insects sampled in these ecotopes, the diversity of identified food sources includes all groups of animals which were described. The presence of *T. brasiliensis* in the most diverse peridomicile ecotopes and the variety of food sources confirm its wide distribution and dispersion capacity, mainly in adults. *Triatoma infestans* females can disperse by walking; such behavior reflects an adaptive strategy to colonize new structures because, unlike flight dispersion, walking allows migration with many eggs in oviducts or dispersion of heavy insects, in good nutritional condition, with difficulty in flying [75]. Furthermore, in this context another possibility for the dispersion of females is the “group” effect [76]. According to this author, the food factor is not the determining factor for migration in females, but rather the reduction of fecundity and oviposition when they are isolated [76].

Nymphs, in turn, presented greater feeding diversity when compared to adult insects, and they showed opportunism and eclecticism. Valença-Barbosa et al. [49] found results similar to the present results regarding the importance of roofing tiles and firewood in peridomicile ecotopes and the detection of DNA of goats in anthropic and wild environments.

A systematic review conducted by Rabinovich et al. [45] indicated that host accessibility is an important factor that shapes the search patterns of blood by triatomines, influenced by the habitats that they colonize. Species which can colonize wild, peridomicile and domestic environments are associated with the more frequent feeding on mammals. For *T. brasiliensis*, humans and rodents are the main food sources [45].

The distribution of genetic strains in domestic and wild cycles of *T. cruzi* seems to be different, considering the various geographical regions. TcI is widely distributed in the Americas in association with the Didelphidae, and it predominates in the domestic cycles of transmission that occur in the north of Amazon Basin. TcII, TcV and TVI predominate in domestic habitats in the Southern Cone of South America. TcIII and TcIV circulate mainly *via* wild transmission cycles [9, 10].

In Brazil, while the TcII lineages and TcI circulate more abundantly in the wild and domestic environments, respectively, TcIII is usually associated with the wild cycle of the parasite. However, the presence of TcIII in vectors and reservoirs of the domestic cycle is considered to be rare. Thus, studies conducted in Ceará [47, 49, 51], Piauí [72, 77, 78], Rio Grande do Norte [78–80] and Bahia [78] have shown the presence of the strains TcI and TcIII in the Caatinga, either in triatomines, mainly *T. brasiliensis* and *P. lutzi*, or in wild hosts such as *Didelphis albiventris* and *Thrichomys apereoides laurentius* or still in domestic hosts such as dogs, rodents and humans. Camara et al. [80] showed an overlap of TcII-related wild and domestic TcII cycles in *T. brasiliensis* in Rio Grande do Norte State, as well as their ability to maintain TcII and TcIII in wild cycles and the emerging risk of introduction of these populations in the domestic cycle.

In this study, a sample of TcIII was characterized from *Monodelphis domestica* caught in the wild environment [47]. Thus, understanding the correlations between DTUs, geographical distribution, habitat, ecology, host species and pathogenicity is still controversial, i.e. the eco-epidemiology of *T. cruzi* is far from being well understood [81].

The occurrence of different DTUs in distinct environments highlights the overlap of wild, peridomicile and intradomicile cycles of *T. cruzi*. The analysis of populations of *T. cruzi* described by environment and time of capture shows that the association of this parasite with *T. brasiliensis* is wide, and it is present in all periods and ecotopes assessed, as demonstrated by the various clusters evidenced for the wild and anthropic environments. The occupation of land by humans, the indiscriminate use of natural resources and availability of ecotopes in the peridomicile directly intervene in approximation and overlap of wild and domestic cycles of *T. cruzi*, through the mobility of triatomines and small mammals, such as rodents and marsupials.

In addition, the identification of important peridomicile ecotopes, e.g. roofing tiles, bricks and stones in the study region, is relevant to the development of appropriate strategies for vector control. Thus, the reduction of the colonies of *T. brasiliensis* in this environment can be decisive for a reduction of the intense movement of *T. cruzi* among existing animals. Furthermore, the specificities of the epidemiological profiles of each region and the influence of environmental, socioeconomic and cultural factors should be considered in order to promote successful control initiatives.

## Conclusions

The significant feeding eclecticism of *T. brasiliensis* and its wide circulation between anthropic and domestic environments demonstrate its epidemiological relevance in maintaining the transmission dynamics of *T. cruzi* in the Caatinga in Ceará. It should be noted that wild, peridomicile and intradomicile cycles of *T. cruzi* may overlap as a result of the association among *T. brasiliensis*, rodents and ruminants, and also because of the presence of TcI and TcIII in intradomiciles in study region. In this context, there is an obvious need to maintain vector control, and successful interventions should consider regional eco-epidemiological differences, regularity and quality of initiatives taken in order to prevent the transmission of *T. cruzi* to humans in the domestic environment.

## Additional files

Additional file 1: The form used in domicile triatomines study. (TIF 1221 kb)

Additional file 2: **Table S1.** Number of captured triatomines, examined and parasitized by tripanosomatids, according to capture place, developmental stage in Tauá municipality (CE) from 2009 to 2015. **Table S2.** Distribution of samples of *Triatoma brasiliensis*, according to capture place, developmental stage and identification of food source in Tauá municipality (CE), 2015. **Table S3.** Samples of *Triatoma brasiliensis*, according to identification of food source, percentage identity and GenBank ID, Tauá municipality (CE), 2015. **Table S4.** Details of *Trypanosomas cruzi* samples caracterized in Tauá municipality (CE) from 2009 to 2015. (XLSX 37 kb)

## Abbreviations

BLASTn: Basic local alignment search tool – nucleotide
*cox*2: Cytochrome oxidase II
HU: Housing unit
IRR: René rachou institute
LIT: Liver infusion tryptose
SESA-CE: Ceará state health secretariat
SL-IL: Mini-exon intergenic spacer
