# Supplementary figures and images for: Triatoma brasiliensis Neiva, 1911: food sources and diversity of Trypanosoma cruzi in wild and artificial environments of the semiarid region of Ceará, northeastern Brazil
Source: Parasit Vectors. 2018 Dec 17;11:642. doi: 10.1186/s13071-018-3235-4 (PMC6296072; doi:10.1186/s13071-018-3235-4)

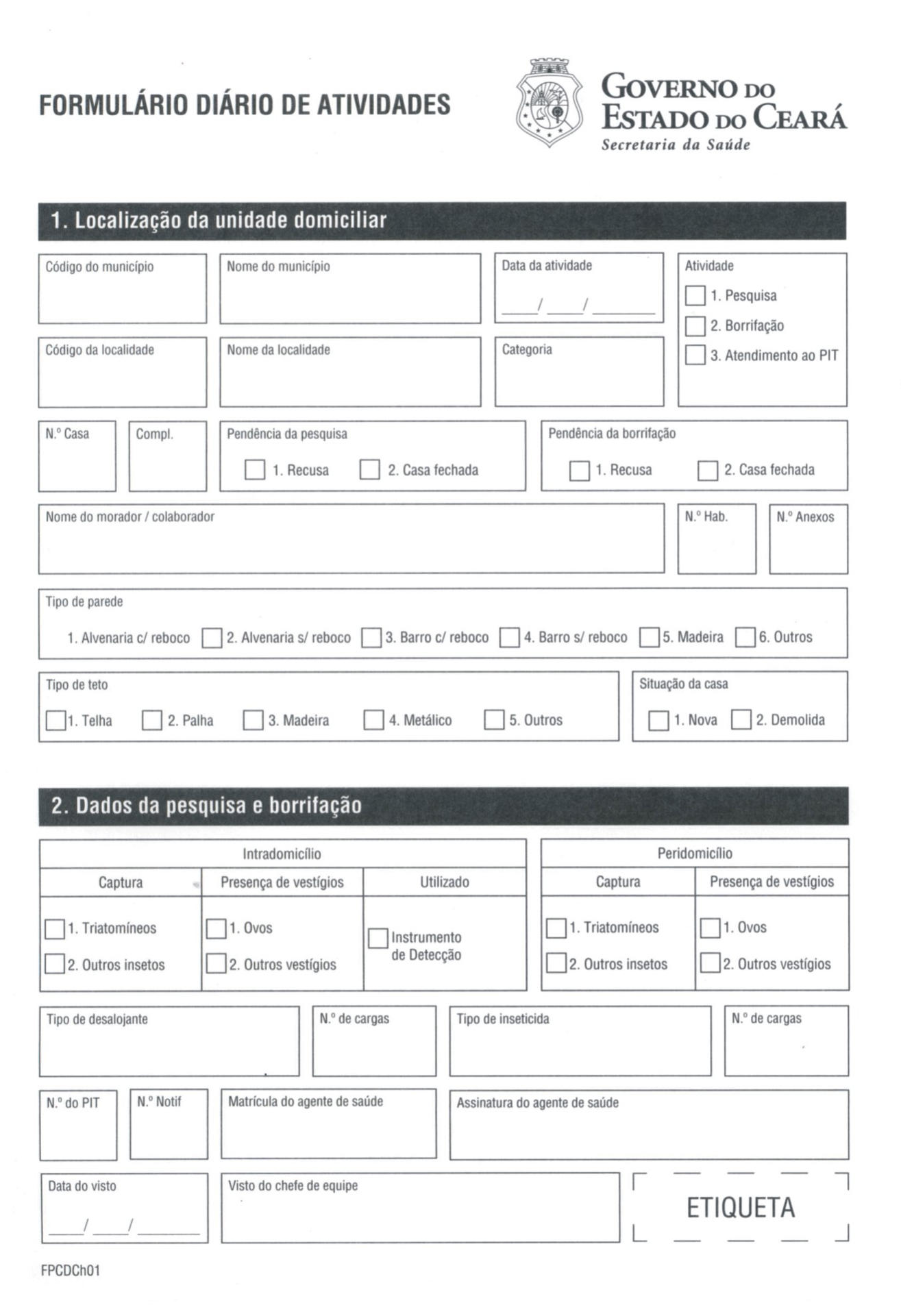

Supplement: Supplementary file 1 — The form used in domicile triatomines study. (TIF 1221 kb) [file 13071_2018_3235_MOESM1_ESM.tif]
